# Supplementary material for: Cloud BioLinux: pre-configured and on-demand bioinformatics computing for the genomics community
Source: BMC Bioinformatics. 2012 Mar 19;13:42. doi: 10.1186/1471-2105-13-42 (PMC3372431; doi:10.1186/1471-2105-13-42)
Supplement: Additional file 1 — Supplementary 1 Cloud BioLinux software documentation in the form of a mini, self-contained website. Users need to download and uncompress the .zip file, and open through a web browser the "index.html" file available on the main directory. (ZIP 1823 kb). [file 1471-2105-13-42-S1.ZIP › Cloud-BioLinux-Package-Documentation/docs/omssa.html]

Bio-Linux Software Documentation Pages

Back to search form

## omssa

|  |  |
| --- | --- |
| Name | omssa |
| Description | **OMSSA** is a free program distributed by the NCBI for analysing and identifying peptides from tandem mass spectrometry peptide spectra. |
| Homepage | http://pubchem.ncbi.nlm.nih.gov/omssa/ |
| Remote Documentation | http://pubchem.ncbi.nlm.nih.gov/omssa/help.htm   http://pubchem.ncbi.nlm.nih.gov/omssa/faq.htm |

|  |
|  |
